# GPCR/endocytosis/ERK signaling/S2R is involved in the regulation of the internalization, mitochondria-targeting and -activating properties of human salivary histatin 1

**DOI:** 10.1038/s41368-022-00181-5

**Published:** 2022-08-15

**Authors:** Dandan Ma, Wei Sun, Cuicui Fu, Kamran Nazmi, Enno C. I. Veerman, Richard T. Jaspers, Jan G. M. Bolscher, Floris J. Bikker, Gang Wu

**Affiliations:** 1 Department of Oral Biochemistry, Academic Centre for Dentistry Amsterdam（ACTA）, University of Amsterdam and Vrije Universiteit Amsterdam, Amsterdam, Netherlands; 2 School of Stomatology, Zhejiang Chinese Medical University, Hangzhou, China; 3 Laboratory for Myology, Department of Human Movement Sciences, Faculty of Behavioral and Movement Sciences, Amsterdam Movement Sciences, Vrije Universiteit Amsterdam, Amsterdam, Netherlands; 4 Department of Oral Cell Biology, Academic Centre for Dentistry Amsterdam（ACTA）, University of Amsterdam and Vrije Universiteit Amsterdam, Amsterdam, Netherlands; 5 Department of Oral and Maxillofacial Surgery/Pathology, Amsterdam UMC and Academic Center for Dentistry Amsterdam （ACTA）, Vrije Universiteit Amsterdam, Amsterdam Movement Science, Amsterdam, Netherlands

**Keywords:** Target identification, Peptides, Protein transport

## Abstract

Human salivary histatin 1 (Hst1) exhibits a series of cell-activating properties, such as promoting cell spreading, migration, and metabolic activity. We recently have shown that fluorescently labeled Hst1 (F-Hst1) targets and activates mitochondria, presenting an important molecular mechanism. However, its regulating signaling pathways remain to be elucidated. We investigated the influence of specific inhibitors of G protein-coupled receptors (GPCR), endocytosis pathways, extracellular signal-regulated kinases 1/2 (ERK1/2) signaling, p38 signaling, mitochondrial respiration and Na+/K+-ATPase activity on the uptake, mitochondria-targeting and -activating properties of F-Hst1. We performed a siRNA knockdown (KD) to assess the effect of Sigma-2 receptor (S2R) /Transmembrane Protein 97 (TMEM97)—a recently identified target protein of Hst1. We also adopted live cell imaging to monitor the whole intracellular trafficking process of F-Hst1. Our results showed that the inhibition of cellular respiration hindered the internalization of F-Hst1. The inhibitors of GPCR, ERK1/2, phagocytosis, and clathrin-mediated endocytosis (CME) as well as siRNA KD of S2R/TMEM97 significantly reduced the uptake, which was accompanied by the nullification of the promoting effect of F-Hst1 on cell metabolic activity. Only the inhibitor of CME and KD of S2R/TMEM97 significantly compromised the mitochondria-targeting of Hst1. We further showed the intracellular trafficking and targeting process of F-Hst1, in which early endosome plays an important role. Overall, phagocytosis, CME, GPCR, ERK signaling, and S2R/TMEM97 are involved in the internalization of Hst1, while only CME and S2R/TMEM97 are critical for its subcellular targeting. The inhibition of either internalization or mitochondria-targeting of Hst1 could significantly compromise its mitochondria-activating property.

## Background

The histatin (Hst) peptide family, which comprises at least 12 small, cationic and histidine-rich peptides, is mainly found in the saliva of higher primates.^1^ According to their functional characteristics, the Hst family is divided into two major groups: (1) antimicrobial Hsts (e.g., Hst5)^2–5^ and (2) cell-activating Hsts (e.g., Hst1 and Hst2).^6–9^ Hst1 and Hst2 can promote a large number of cell activities, such as adhesion, migration, cell-cell adhesion, differentiation and angiogenesis.^6–12^ With these functions, Hsts play important roles in protecting oral mucosa and maintaining its homeostasis.^8,11,13^

Previous findings indicate that the cell-activating effects of Hst1 and Hst2 can be abolished by both the specific inhibitors of G protein-coupled receptors (GPCR) and extracellular signal-regulated kinases 1/2 (ERK1/2) pathway.^7,14^ Moreover, our recent study has shown that within 5 min after administration to cells, both Hst1 and Hst2 are quickly taken up and targeted to mitochondria and endoplasmic reticulum (ER) in vitro.^9^ Furthermore, our findings suggest that Hst1 and Hst2 but not Hst5 activate mitochondrial energy production so as to enhance metabolic activity of different types of cells.^9^ Similarly, Hst5 exerts antimicrobial activity upon its uptake and targeting to mitochondria in fungi cells.^15^ In contrast, the D-enantiomer of Hst2 (D-Hst2) shows no internalization by cells and does not promote the migration of epithelial cells.^14^ Interestingly, a recent study shows that the siRNA KD of Sigma-2 receptor (S2R)/Transmembrane Protein 97 (TMEM97) significantly compromises the internalization and pro-migratory effect of Hst1.^16^ All these findings strongly suggest a critical role of internalization and mitochondria-targeting in Hsts’ biological functions. However, the signaling pathways regulating Hsts’ internalization as well as their mitochondria-targeting and -activating properties remain to be further elucidated.

Passive and energy-independent penetration seems less plausible for the internalization of Hst1 as the uptake of Hst1 is inhibited at 4 °C,^7^ which suggests that the active and energy-dependent endocytosis are involved in the uptake of Hst1. In line with this finding, a recent study shows that the endosomal Ras and Rab interactor 2 (RIN2)/Rab5/Rac1 signaling axis regulates Hst1-induced endothelial cell migration.^6^

Endocytosis is an active and energy-dependent transportation process for macromolecules into a cell in vesicles or vacuoles pinched off of the plasma membrane.^17^ With regard to endocytosis, this is generally divided into two major categories are distinguished: phagocytosis and pinocytosis.^18^ Phagocytosis occurs in specialized immune cells (e.g., macrophages, monocytes, and neutrophils) and functions to engulf and digest large particles, such as cellular debris and pathogens.^19^ Phagocytosis can form intracellular phagosomes.^19^ In contrast, pinocytosis occurs in a broad variety of cells, functions to take up fluids and small-size solutes, and forms endosomes. Furthermore, pinocytosis can be classified into at least four mechanisms: macropinocytosis, clathrin-mediated endocytosis (CME), caveolae-mediated endocytosis, and clathrin- and caveolae-independent endocytosis.^20–22^ It remains to be elucidated which endocytosis pathways are responsible for Hst1´s uptake and how they influence Hst1´s mitochondria-targeting and -activating properties.

In the present study, we investigated the influence of specific inhibitors of GPCR, various endocytosis pathways, ERK signaling, p38 signaling, an inhibitor of mitochondrial respiration, and an inhibitor of Na^+^/K^+^-ATPase activity as well as siRNA knockdown (KD) of S2R/TMEM97 on the mitochondria-targeting and -activating properties of Hst1. We adopted live imaging and, for the first time, monitored the whole intracellular trafficking process of Hst1 through endosomes to mitochondria.

## Results

### The uptake of Hst1 by HO1N1 cells was a stereospecific and energy-dependent process

**Fig. 1:**
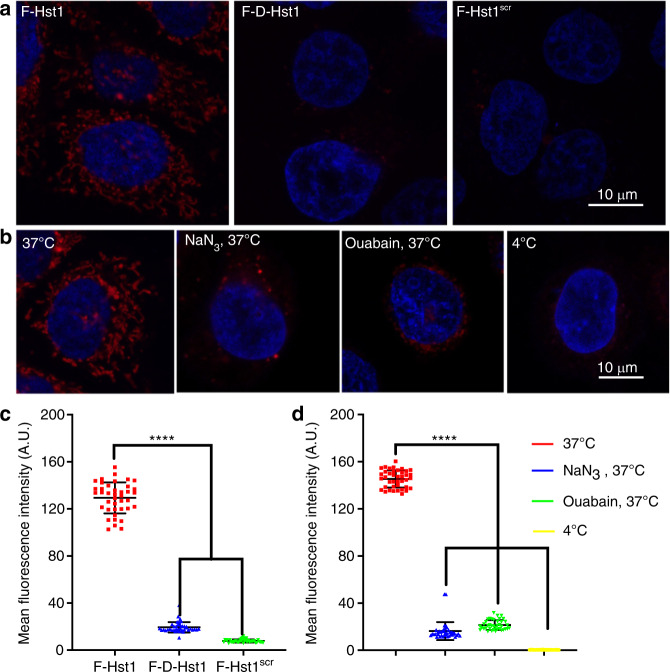
Structural requirements and energy-dependence for the uptake of Hst1. **a** HO1N1 cells were pre-incubated with 2 μmol·L^−1^ F-Hst1 and its variants for 60 min, then nuclear DNA was stained with NucBlue™ live cell regent for 20 min. Representative a confocal laser scanning microscopy (CLSM) images of internationalization of F-Hst1, F-D-Hst1 and F-Hst1scr in HO1N1 cells. **b** Energy dependence experiments were performed by cooling the cells at 4 °C for 60 min to minimize cellular activity. Subsequently, cells were incubated with metabolic inhibitors 20 μmol·L^−1^ NaN_3_ (inhibitor of cytochrome C oxidase) and 50 μmol·L^−1^ ouabain (inhibitor of Na^+^/K^+^-ATPase) at 37 °C for 60 min. Then cells were incubated with 2 μmol·L^−1^ F-Hst1 for 60 min and stained with NucBlue™ live cell reagent for 20 min to stain DNA. Representative images of internationalization of F-Hst in HO1N1 cells at 4 °C cultured in medium with NaN_3_ or with ouabain with CLSM Bar = 10 μm, F-Hst1 (in red); nuclei (in blue). **c** Mean intracellular fluorescence intensity of F-Hst1, F-D-Hst1 and F-Hst1scr was quantitated using Fiji. **d** Mean intracellular fluorescence intensity of F-Hst1 was quantitated using Fiji software. Data are presented as mean ± SD (*n* = 40); ****P < 0.000 1

To establish whether the cellular uptake of F-Hst1 in HO1N1 cells was a structurally required process, cells were incubated with F-Hst1, F-D-Hst1, and F-Hst1^scr^. 60 min post-incubation, fluorescence intensities of F-D-Hst1 and F-Hst1^scr^ in intracellular space were much weaker compared to that of F-Hst1 (Fig. 1a. To establish whether the cellular uptake of F-Hst1 in HO1N1 cells was an energy-dependent process, cells were pre-incubated in three conditions: 1) at 4 °C, 2) in the presence of NaN_3_, and 3) in the presence of ouabain at 37 °C. CLSM images revealed that the intracellular accumulation of F-Hst1 was completely abolished at 4 °C (Fig. 1b). Quantitative analysis showed that the fluorescence intensity per cell of F-D-Hst1 and F-Hst1^scr^ was significantly lower (only 15% and 6% respectively) compared to that of F-Hst1 (Fig. 1c). The fluorescence intensity of F-Hst1 per cell was significantly decreased by NaN_3_ and ouabain (about 90% and 85%, respectively) (Fig. 1d).

### The uptake, subcellular targeting and promoting effect of Hst1 on cell metabolic activity were regulated by GPCR and ERK signaling

**Fig. 2:**
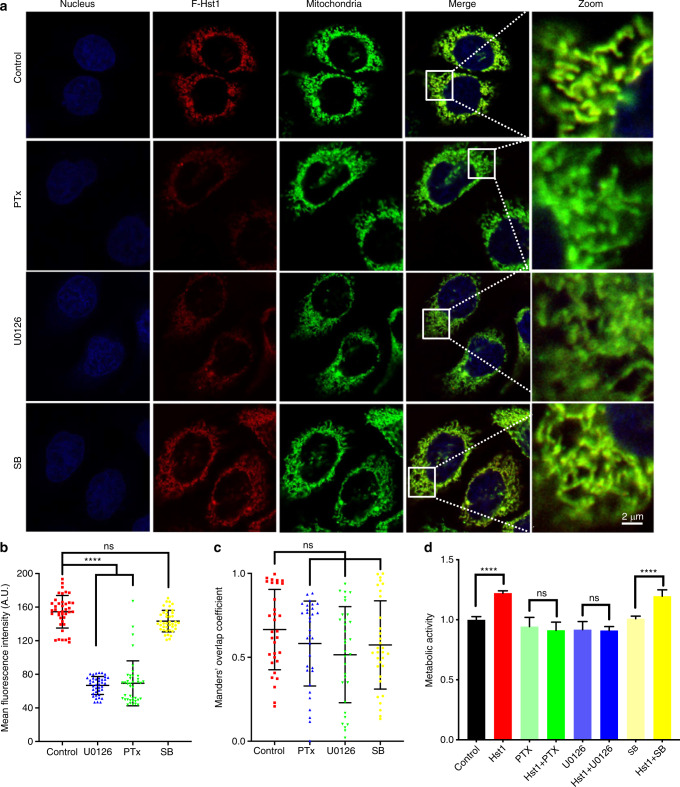
The uptake, subcellular targeting, and promoting effect of Hst1 on cell metabolic activity are regulated by GPCR and ERK signaling. Experiments to assess Hst1 function dependence on GPCR, ERK or p38 MAPK signaling were performed by pre-incubating HO1N1 cells with specific inhibitors. Then cells were incubated with or without 2 μmol·L^−1^ F-Hst1 for 60 min and stained with NucBlue™ live cell reagent. **a** Typical CLSM images of subcellular localization of F-Hst1 in HO1N1 cells under the treatment with 100 ng·mL^−1^,PTx, 10 μmol·L^−1^ U0126, or 10 μmol·L^−1^ SB (the inhibitors of GPCR, ERK1/2 activity, and p38MAPK, respectively). Bar = 2 μm. F-Hst1 (in red); nuclei (in blue); mitochondrial (in green). **b** Mean intracellular fluorescence intensity of F-Hst1 was quantitated using Fiji software. **c** Mander’s overlap coefficients of F-Hst1 with mitochondria in the presence of PTx or U0126. **d** Cells were then incubated in the presence or absence of various inhibitors and 10 μmol·L^−1^ Hst1 for 60 min. Untreated cells were used as control. The PrestoBlue solution (10 μL) was added into each well after 30 min, after which absorbance was measured at wavelengths 570 nm excitation and 600 nm emission. Graph depicting the effect of 100 ng·mL^−1^, 10 μmol·L^−1^ U0126, and 10 μmol·L^−1^ SB on Hst1-stimulated cellular metabolic activity was measured by the PrestoBlue assay. Data are presented as mean ± SD; ****P < 0.000 1. ns not significant

To determine the role of GPCR and ERK signaling in the Hst1-cell interactions, we assessed the uptake, subcellular targeting, and promoting effect on cell metabolic activity of Hst1 in the presence of specific inhibitors of Gαi/o subunits of the G protein (Pertussis Toxin, PTx), ERK signaling (U0126), p38 signaling (SB203580) (Fig. 2). The CLSM images showed that both PTx and U0126 could significantly decrease the uptake of F-Hst1 (Fig. 2a). Quantitative analysis showed that the fluorescence AU of Hst1 in PTx and U0126-treated cells were significantly decreased by about 56% in comparison to the control (Fig. 2b). Mander’s overlap coefficients were measured to describe the co-localization of the F-Hst1(red) with mitochondria (green). Mander’s overlap coefficients of the cells treated with PTx, U0126 and SB203580 were 0.58 ± 0.25, 0.52 ± 0.28 and 0.57 ± 0.26, which still indicated significant co-localization. Compared with the value of the control group (0.67 ± 0.23), there are no significant differences among these groups (Fig. 2c). U0126 and PTx completely abolished the stimulating effects of Hst1 on the energy metabolism of HO1N1 cells (Fig. 2d). In contrast, SB203580 had no effect, neither on the cellular uptake, nor on the histatin-meditated stimulation of the energy metabolism (Fig. 2).

### The uptake, subcellular targeting and promoting effect of Hst1 on cell metabolic activity were regulated by specific endocytic pathways

**Fig. 3:**
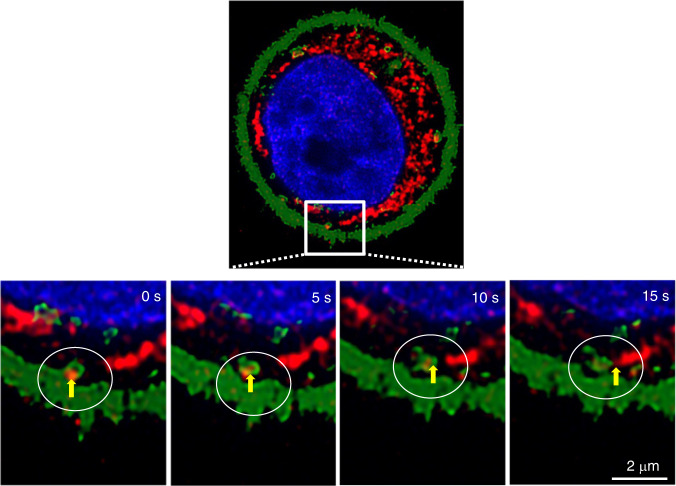
Trafficking and intracellular distribution of F-Hst1. Before adding F-Hst1 to the cell culture medium, nuclear DNA was stained with NucBlue™ live cell reagent. The cell membrane was labeled using PKH67 Green Fluorescent Cell Linker Kit. Representative CLSM images showed that an endocytic vesicle was formed from the beginning to the later time points throughout the very fast translocation process (as indicated in the yellow arrow). After uptake, the loaded vesicle pinched off from the cell membrane and was released into the cytoplasm. Bar = 2 μm. F-Hst1 (in red); nuclei (in blue); cell membrane (in green)

**Fig. 4:**
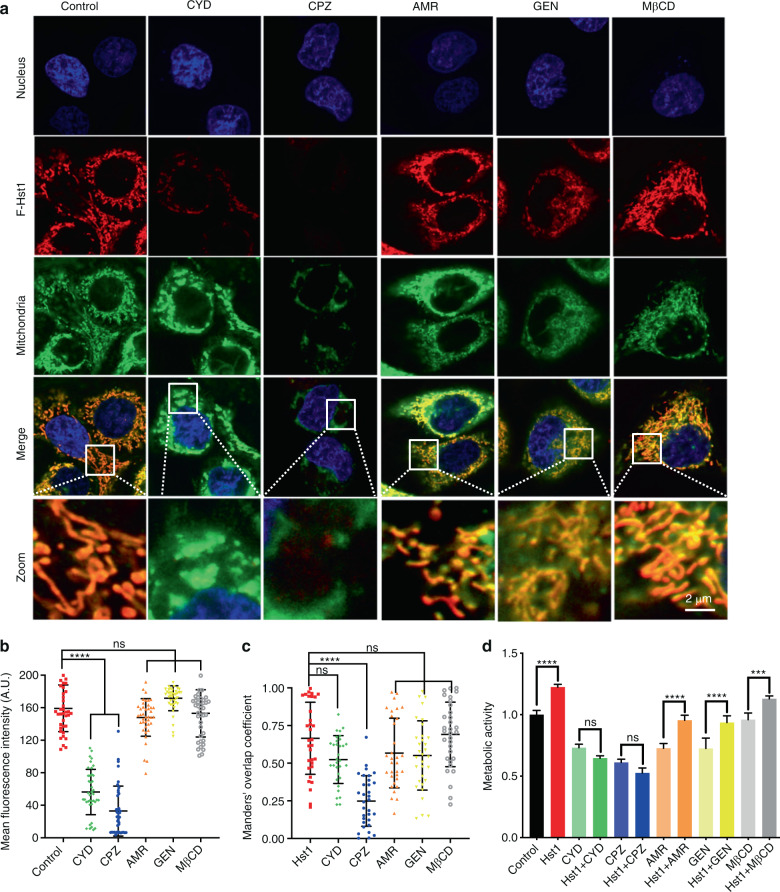
The uptake, subcellular targeting and promoting effect of Hst1 on cell metabolic activity of Hst1 were regulated by specific endocytic pathways. To analyze the potential mechanism on uptake pathways of Hst1, HO1N1 cells were pre-incubated with five types of endocytic inhibitors including cytochalasin D (CYD, inhibitor of phagocytosis), chlorpromazine (CPZ, inhibitor of clathrin-mediated endocytosis), amiloride (AMR, inhibitor of macropinocytosis mediated endocytosis), genistein (GEN, inhibitor of caveolae-mediated endocytosis) and methyl-β-cyclodextrin (MβCD, inhibitor of lipid raft mediated endocytosis). Before imaging, nuclear DNA was stained with NucBlue™ live cell reagent. For the detection of mitochondria and ER, cells were stained with MitoTracker® Green FM and ER-Tracker Blue-White DPX. **a** Typical images of F-Hst1 distribution within HO1N1 cells after pre-treatment with endocytosis inhibitors: cytochalasin D, genistein and chlorpromazine, methyl-β-cyclodextrin, amiloride. Bar = 2 μm. F-Hst1 (in red); nuclei (in blue); mitochondrial (in green). **b** Relative intracellular fluorescence intensity of F-Hst1 was quantitated using Fiji software. **c** Mander’s overlap coefficients of F-Hst1 with mitochondria in the presence of endocytosis inhibitors: cytochalasin D, genistein and chlorpromazine, methyl-β-cyclodextrin, amiloride. **d** Cells were then incubated in the presence or absence of various inhibitors and Hst1 for 60 min. Untreated cells were used as control. The PrestoBlue solution was added into each well after 30 min, after which absorbance was measured at wavelengths 570 nm excitation and 600 nm emission. Graph depicting the effect of various endocytosis inhibitors on effects of Hst1 on the cellular metabolic activity of HO1N1 human epithelial cells was measured by the PrestoBlue assay. Data are presented as mean ± SD; ***P < 0.001, ****P < 0.000 1. ns not significant, CYD cytochalasin D, CPZ chlorpromazine, AMR amiloride, GEN genistein, MβCD methyl-β-cyclodextrin

Since the uptake of Hst1 seems to be an energy-dependent process, we further identified the role of endocytosis in mediating the intracellular trafficking and subcellular targeting of Hst1. Time-lapse images revealed that the F-Hst1 was entrapped in an endocytic vesicle with its membrane derived from a cell membrane using PKH-67 (Fig. 3). Then the F-Hst1-containing vesicle quickly pinched off from the cell membrane and was directly transferred to an F-Hst1-enriched mitochondrion-like structure (Fig. 3). We aimed to further unravel the role of specific endocytic pathways. For this purpose, several specific inhibitors, including CYD, GEN, CPZ, MβCD and AMR were applied. The CLSM images revealed that the fluorescence intensity of F-Hst1 per cell under the treatments with CYD (56.26 ± 27.70) A.U. and CPZ (32.92 ± 30.46) A.U. were significantly lower than that of the control (159.21 ± 28.50) A.U. (Fig. 4a, b), suggesting that the phagocytosis and CME pathways played a critical role for the uptake of the F-Hst1 by human epithelial cells. The Mander’s overlap coefficient of the CYD group (0.52 ± 0.02) showed no significant difference with that of the control group (0.67 ± 0.23) (Fig. 4c). In comparison, the treatment with CPZ dramatically reduced Mander’s overlap coefficients to 0.24 ± 0.16 (Fig. 4c). These findings suggested that CME pathways were involved in the subcellular targeting of F-Hst1 (Fig. 4c). In contrast, treatments with genistein, MβCD, or AMR did not significantly affect the uptake and subcellular targeting of F-Hst1 (Fig. 4). Similar to these findings, the treatments with CYD or CPZ completely nullified the promoting effect of Hst1-on the metabolic activity of human epithelial cells, while other inhibitors failed to do so (Fig. 4d).

### Early endosome (EE) but not late endosome (LE) was involved in intracellular trafficking of F-Hst1

**Fig. 5:**
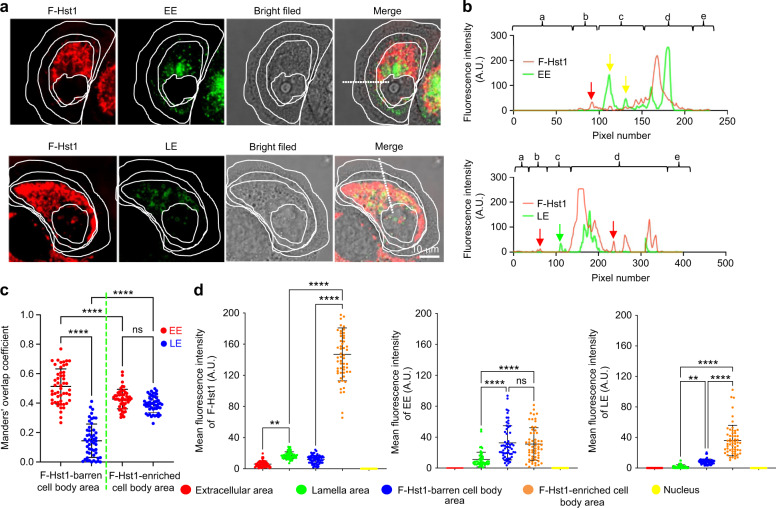
Confocal images show F-Hst1 co-localization with GFP-Rab5a-tagged early endosome (EE) but not with GFP-Rab7a-tagged late endosome (LE). Before adding 2 μmol·L^−1^ F-Hst1 to the cell culture medium, EE and late LE were labeled using Invitrogen™/CellLight™ EE-GFP and Invitrogen™/CellLight™ LE-GFP, respectively, following the manufacturer’s protocols. **a** Typical CLSM images of HO1N1 cells transfected with GFP-Rab5a-tagged EE or GFP-Rab7a-tagged late LE. Bar = 10 μm. F-Hst1 (in red); EE (in green); LE (in green). **b** Fluorescence intensity profile of regions of interest (white line in A). **c** Manders’ overlap coefficients of F-Hst1 with EE and LE. **d** Mean intracellular fluorescence intensity of F-Hst1, EE and LE were quantitated using Fiji software. a: extracellular area; b: lamella area; c: F-Hst1-barren cell body area; d F-Hst1-enriched cell body area; e: Nucleus; Data are presented as mean ± SD (*n* = 58); **P < 0.01, ****P < 0.000 1. ns not significant

**Fig. 6:**
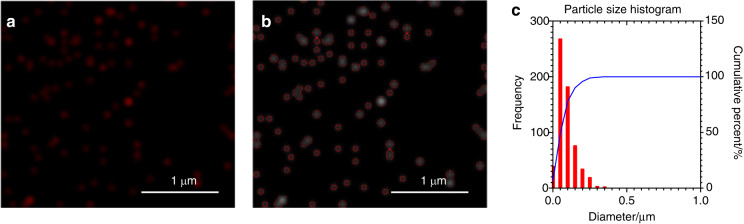
Confocal image and particle size distribution histogram of F-Hst1. HO1N1 cells were pre-incubated with 2 μmol·L^−1^ F-Hst1 for 60 min. **a** Representative CSLM images of F-Hst1-associated particle. **b** Outlines of F-Hst1 associated particle. **c** The respective histogram of F-Hst1 associated particle size distribution. Bar = 1 μm

Based on the above observations, we further identified the potential roles of EE and LE in the intracellular trafficking and subcellular targeting activities of Hst1 were investigated. For this purpose, we first identified the distribution pattern of F-Hst1 and EE or LE that were indicated by GFP-fused Rab5a or GFP-fused Rab7a respectively in intracellular space (Fig. 5). In the direction from the extracellular space to nucleus, within a spread cell 3 parts can be distinguished: (1) lamella, (2) cell body, and (3) nucleus (Fig. 5a). In the lamella area (outside the border of the cell body), Hst1 distribution showed a radial and linear distribution pattern and the fluorescence intensity of F-Hst1 was significantly lower than that of the Hst1-enriched cell body area but much higher than that of Hst1-barren cell body area (Fig. 5a). No LE could be detected in the lamella area. EE showed significant co-localization with the F-Hst1 in the cell body area (yellow arrows, Fig. 5b). Thereafter, we identified the co-localization of Hst1 with either EE or LE in the Hst1-barren cell body area (Fig. 5b, c), where Hst1 was transported and targeted to mitochondria. A significant co-localization between F-Hst1 and EE was detected in this area with the Manders’ overlap coefficient value at 0.51 ± 0.14 (Fig. 5c). In contrast, no significant co-localization was found between F-Hst1 and LE as the Manders’ co-localization coefficient value (0.14 ± 0.12) was much lower than 0.5 (Fig. 5c). As above described, in the perinuclear space of the cell body, the highlighted, F-Hst1-enriched mitochondria-like structures formed a F-Hst1-enriched cell body area, the average fluorescence intensity of F-Hst1 reached 146.76 ± 33.82  A.U. (Fig. 5d). Many EE and LE vesicles showed scattered distribution without a certain pattern in this area with average fluorescent intensities of GFP-fused Rab5a or GFP-fused Rab7a at 31.057 ± 20.99 A.U., and 35.97 ± 19.94 A.U., respectively (Fig. 5d). The density of LE in this area was quite lower than that in the Hst1-enriched cell body area. However, there were no significant differences in density of the EE between Hst1-barren cell body areas and Hst1-enriched cell body areas (Fig. 5d). In the space between the mitochondria-enriched area and the border of the cell body was an Hst1-barren cell body area with the average fluorescence intensity as low as (11.28 ± 5.33) A.U. (Fig. 5d). In this area, the particles of F-Hst1 presented a relatively uniform spherical shape with their diameter ranging from 16 to 175 nm (Fig. 6).

**Fig. 7:**
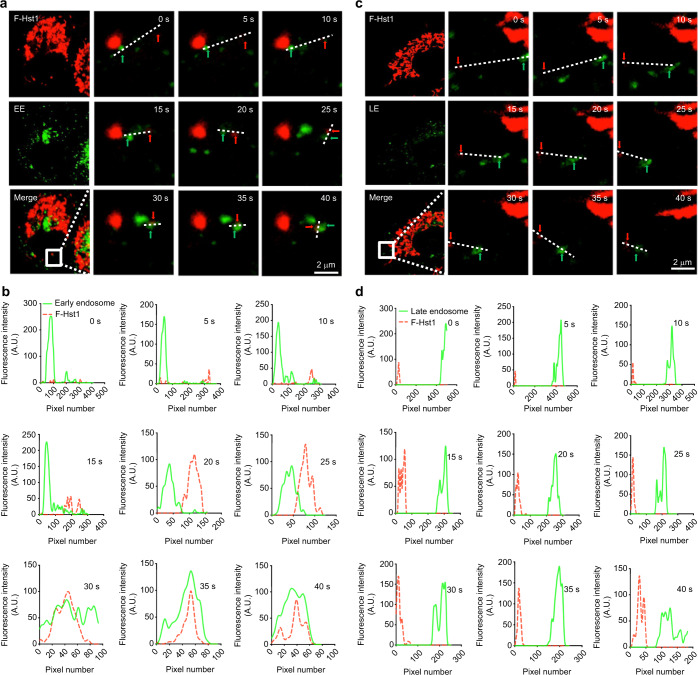
Time-lapse fluorescent microscopy reveals that the early endosome-F-Hst1 fusion takes place in HO1N1 cells in a short time. Before adding 2 μmol·L^−1^ F-Hst1 to the cell culture medium, EE and LE were labeled using Invitrogen™/CellLight™ EE-GFP and Invitrogen™/CellLight™ LE-GFP, respectively. **a** Representative CSLM images of F-Hst1 interaction with EE. Bar = 2 μm. F-Hst1 (in red); EE (in green). **b** Fluorescence intensity profile of regions of interest (white line in (**a**)). **c** Representative images of F-Hst1 interaction with LE. Bar = 2 μm. F-Hst1 (in red); LE (in green). **d** Fluorescence intensity profile of regions of interest (white line in (**c**)). Red arrow and green arrow indicate the F-Hst1 and endosome, respectively

Thereafter, we used the time-lapse imaging functions of the confocal microscopy with an interval of 5 s to monitor the association process of F-Hst1 with GFP-Rab5a-labeled EEs (Fig. 7). Live imaging showed that GFP-Rab5a-labeled EEs gradually approached (from 0 to 15 s) and touched (at 20 s) F-Hst1, finally fusing (from 25 to 45 s) to form a Hst1/EE complex (Fig. 7a, b). In contrast, nearly no such approaching, touching and fusion processes were detected between Hst1 and LE (Fig. 7c, d).

**Fig. 8:**
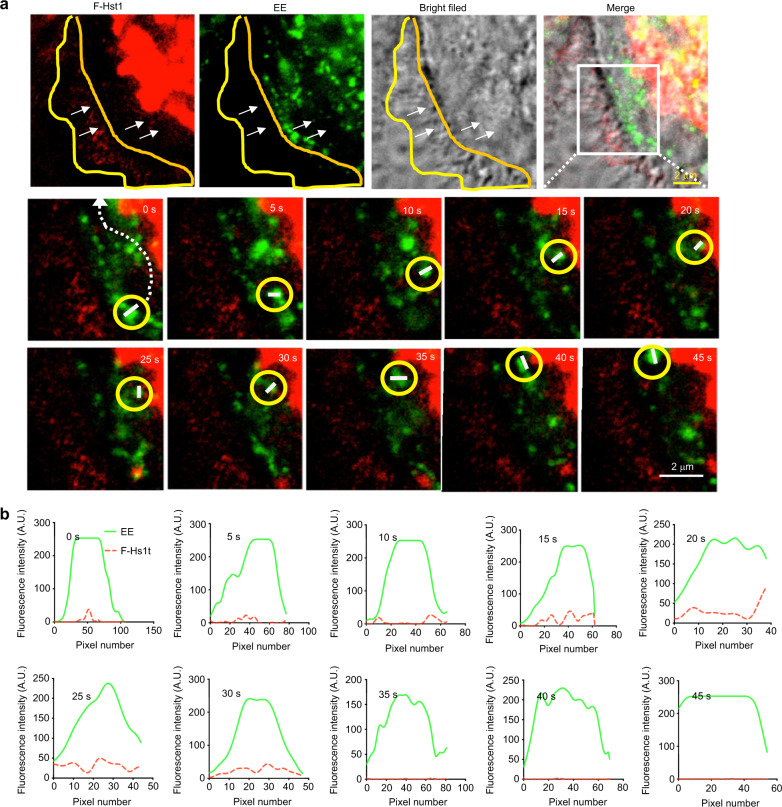
F-Hst1-Endosome-mitochondrion interactions facilitate functional Hst1 transfer in a living cell. Before adding 2 μmol·L^−1^ F-Hst1 to the cell culture medium, early endosome (EE) was labeled using Invitrogen™/CellLight™ Early Endosomes-GFP and 500 nmol·L^−1^ MitoTracker® Green FM following the manufacturer’s protocols. **a** Representative images of F-Hst1 interaction with EE or mitochondrial-like structure. The white arrows indicate that F-Hst1 was bound to the cellular membrane and translocated to the cytoplasm. The area marked by the white box shows an expanded view of the time-lapse of the F-Hst1-Endosome-mitochondrion interactions. The trajectories are marked by the white dotted line in the first enlargement. Bar = 2 μm. F-Hst1 (in red); EE (in green); F-Hst1-targeted mitochondria structure (in red). **b** Fluorescence intensity profile of regions of interest (white dotted line in a)

Next, we monitored the trafficking of an F-Hst1-containing GFP-Rab5a-labeled EE and its spatial association with Hst1-enriched mitochondria-like structure (Fig. 8). The time-lapse images showed that the F-Hst1-containing GFP-Rab5a-labeled EE (yellow circle) moved from the border of the cell body towards the Hst1-enriched cell body area from 0 to 15 s (Fig. 8a, b). Thereafter, the F-Hst1-containing GFP-Rab5a-labeled EE (yellow circle) touched and associated with the Hst1-enriched mitochondria-like structure from 15 to 25 s, during which the F-Hst1 was released to mitochondria (Fig. 8a, b). Then the dissociated GFP-Rab5a-labeled EE containing no F-Hst1 moved back towards the border of the cell body (Fig. 8a, b).

### Endoplasmic reticular protein S2R/TMEM97 regulated the uptake, subcellular targeting, and promoting effect on cell metabolic activity of Hst1

**Fig. 9:**
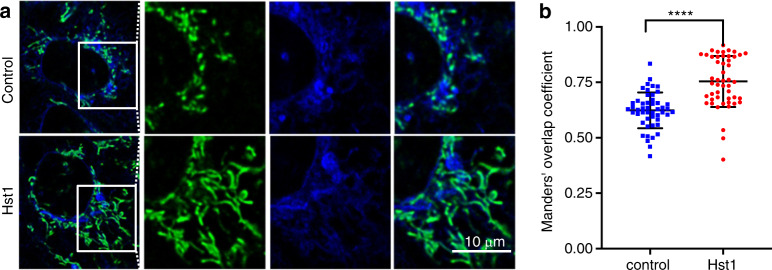
Hst1 promotes ER-mitochondria interaction. The HO1N1 cells were cultured in the presence of 10 μmol·L^−1^ Hst1 for 24 h, and stained with 500 nmol·L^−1^ MitoTracker® Green FM and 500 nmol·L^−1^ ER-Tracker Blue-White DPX. **a** Representative CSLM images of MAM in the control and Hst1-treated HO1N1 cells. Bar = 10 μm. ER in blue; mitochondria in green. **b** Mander’s overlap coefficients of co-localization mitochondria with ER in the control and Hst1-treated HO1N1 cells. Data are presented as mean ± SD; ****P < 0.000 1

**Fig. 10:**
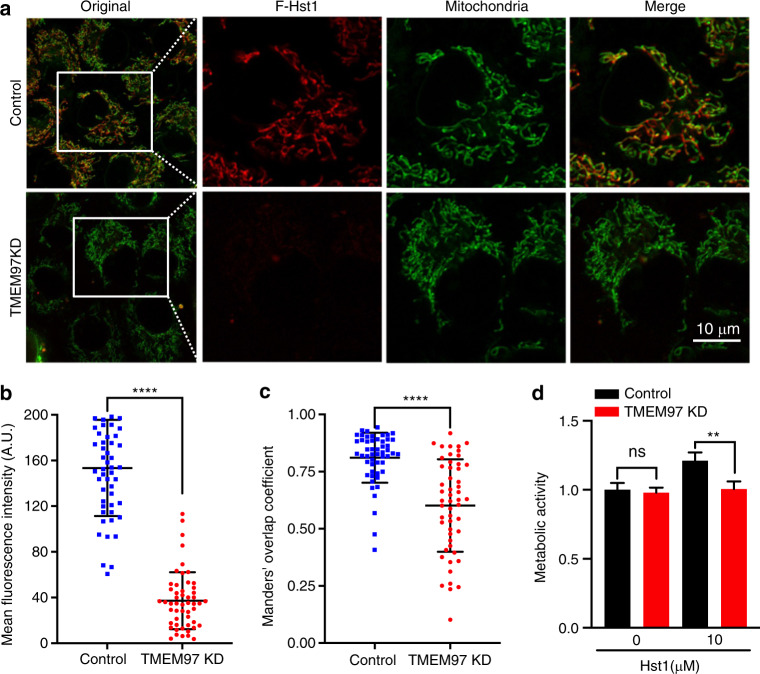
Knockdown (KD) of TMEM97 alter the mitochondrial-targeting and -activating properties of Hst1. HO1N1 cells were transfected with siRNA to downregulate the expression of TMEM97, and subsequently cultured with in medium supplemented with F-Hst1. **a** Typical image of subcellular localization of F-Hst1 with control siRNA transfection and loss of both TMEM97 in HO1N1 cells. Bar = 10 μm. F-Hst1 (in red); mitochondrial (in green). **b** Mean intracellular fluorescence intensity of F-Hst1 was quantitated using Fiji software. **c** Mander’s overlap coefficients of co-localization F-Hst1 with mitochondria in the TMEM97 KD. **d** Effects of TMEM97 on Hst1-stimulated cellular metabolic activity quantified by PrestoBlue assay. Data are presented as mean ± SD; ****P < 0.000 1

In our previous study, we have shown that Hst1 exhibits a high co-localization with both mitochondria and endoplasmic reticulum (ER). Based on those observations, we hypothesized that Hst1 enhances mitochondria-ER contacts (MERCs) and their interactions. In this study, we labeled mitochondria and ER with mitotracker™ and ER-tracker™, respectively, to test this hypothesis. We found that the Mander’s overlap coefficient of mitochondria and ER was significantly enhanced from 0.62 ± 0.08 to 0.75 ± 0.12 by Hst1 (Fig. 9). Hitherto, the targets of Hst1 on mitochondria and ER remain largely unknown. A very recent study shows that ER protein S2R/TMEM is a target of Hst1 on ER. In this study, we showed that the siRNA KD of S2R/TMEM significantly reduced the fluorescence intensity of F-Hst1 per cell by about 76% (Fig. 10). Furthermore, the Mander’s overlap coefficient of Hst1 with mitochondria was also significantly decreased from 0.81 ± 0.10 to 0.60 ± 0.20. The siRNA KD of TMEM97 also nullified the promoting effect of Hst1 on cell metabolic activity.

## Discussion

Hst1 can trigger a series of cell-activating activities, one of which is to activate mitochondrial energy production so as to promote cell metabolic activity.^9,23^ Our recent study suggests that the uptake and mitochondria-targeting are highly important for the mitochondria-activating property of Hst1.^9^ However, hitherto, the internalization pathway, intracellular trafficking processes, mitochondria-targeting of Hst1 and their regulating signaling pathways remain to be elucidated. In this study, by inhibiting cellular respiration we showed the energy-dependent property of Hst1’s uptake. Furthermore, specific inhibitors of GPCR, ERK and phagocytosis also significantly reduced the uptake but not subcellular targeting of Hst1, which was accompanied by the nullification of the promoting effect of Hst1 on cell metabolic activity. In contrast, the specific inhibitor for CME and siRNA KD of S2R/TMEM97 significantly downregulated both the uptake and mitochondria-targeting of Hst1, which also abolished Hst1’s promoting effect on cell metabolic activity. By adopting a time-lapse imaging, we further illustrated the intracellular distribution pattern, trafficking and targeting process of Hst1, during which EE played an important role.

Hst1 and Hst2 are the major wound-closure stimulating factors in human saliva.^7,10,14^ Hst1 is the primary gene product of *HTN1*, and Hst2 is a shorter variant; the result of post-translational proteolysis.^7^ Our previous study has shown that the D-enantiomer of Hst2 was significantly less depleted by epithelial cells than Hst1 and Hst2, suggesting a receptor-specific target for Hst2.^7^ Consistently, using confocal microscopy, our current study further show that the uptake of fluorescently labeled D-Hst1 and Hst^scr^ was significantly less than Hst1, again pointing towards a stereospecific and amino acid sequence-dependent property for Hst1’s cellular uptake (Fig. 1a, b). Furthermore, D-Hst2 and Hst^scr^ do not bear cell-activating effects.^7,8,10,14^ These results highly suggest the important role of cellular uptake in the biological functions of Hst1 and Hst2. Consistent with our previous finding,^7^ we showed that the uptake and intracellular accumulation of Hst1 was significantly inhibited at 4 °C, which showed the energy-dependent property of Hst1’s uptake (Fig. 1c, d). We further corroborated such a conclusion by showing that the uptake of F-Hst1 could be significantly reduced when suppressing mitochondrial ATP production by pre-treating cells with NaN_3_, an inhibitor of cytochrome C oxidase (complex IV)^24^, or with ouabain, an inhibitor of Na^+^/K^+^-ATPase^25^ (Fig. 1b, d). These findings indicate that the uptake of Hst1 was an energy-dependent process, thus excluding the possibility of direct and passive penetration. Concern may be raised whether the peptide bears sufficient stability in intracellular space for the live cell imaging in the current study. In our previous study, we have already shown that Hst1 remains intact up to 2 h in a protease-enriched microenvironment (chronic wound extracts) [3] while our quantification was performed within 1 h. Furthermore, the D-form peptide is widely accepted to be more stable than the corresponding L-form peptide [4]. Therefore, the Hst1 and its variant peptides should have sufficient stability for the intracellular live cell imaging in this study.

GPCRs, also known as seven-(pass)-transmembrane domain receptors and G protein-linked receptors, are a large group of G protein-coupling cell-surface receptors to mediate the cellular responses of molecules outside cells.^26^ Depending on the α subunit type (Gα_s_, Gα_i/o_, Gα_q/11_, Gα_12/13_), the G protein’s α subunit, together with the bound GTP, dissociates from the β and γ subunits to activate a large variety of intracellular signaling pathways.^27^ PTx catalyzes the ADP-ribosylation of the Gα_i_ subunits (Gα_i_, Gα_o_, and Gα_t_ except Gα_z_) of the heterotrimeric G protein, which prevents the G proteins from interacting with G protein-coupled receptors on the cell membrane.^28^ Our previous study shows that the administration of PTx nullifies Hst1-induced cell migration.^14^ It remains unclear whether GPCR is also involved in Hst1’s uptake, mitochondria-targeting, and promoting effect on cell metabolic activity. Our results in the present study show that PTx significantly reduced the uptake of Hst1 but not its co-localization with mitochondria (Fig. 2b, c). Furthermore, PTx also nullified the promoting effect of Hst1 on cell metabolic activity (Fig. 2d). These findings indicate that the GPCR Gα_i_ subunits played an important role in mediating Hst1’s uptake and mitochondria-activating function. Since the inactivation of GPCR by PTx dramatically reduced the uptake of Hst1 by cells, we hypothesized that the uptake of Hst1 might be partially mediated by the GPCR-activated endocytosis that is initiated by the phosphorylation by GPCR kinase (GRK), the binding of arrestins to phosphorylated GPCRs and the binding of components of endocytic lattices (clathrin heavy chain, AP2 alpha subunit and PIP2) to arrestins, thereby promoting agonist-dependent clustering of GPCRs in clathrin-coated pits.^29^ After this clustering, agonist/GPCRs-containing endocytic vesicles are delivered into early endosomes, where ligand dissociation, receptor dephosphorylation and molecular sorting events occur. Receptor sorting into a recycling pathway mediates non-destructive return of receptors to the plasma membrane, which supports sustained cellular responsiveness to the agonist.^29^ The involvement of endocytosis in the uptake of Hst1 is supported by a recent finding that Hst1 promotes the spreading of endothelial cells through the activation of the Ras and Rab interactor 2 (RIN2)/Rab5/Rac1 signaling axis, as Hst1 increases the recruitment of RIN2, a Rab5–guanine nucleotide exchange factor (GEF) to early endosomes, leading to sequential Rab5/Rac1 activation.^6^ Rab5 GTPase is a key regulator in both endocytosis and following membrane trafficking events, such as CME, macropinocytosis, phagocytosis, fusion and motility of early endosomes, and transcriptional regulation.^30,31^ Activated Rab5 further activates Rac1 that regulates cytoskeleton organization to modulate phagocytosis, cell migration, adhesion, and differentiation.^32,33^ The transfection of the dominant-negative form of Rab5 nullifies Hst1-induced Rac1 activation and compromises Hst1-triggered cell migration, which suggests a key role of Rab5 in Hst1’s cell-activating effects as well.^6^ All these findings suggest the involvement of endosomal signaling in Hst1-cell interactions. However, hitherto, there is still a lack of substantial evidence to show the direct interaction between Hst1 and endosomes. In this study, we adopted a time-lapse confocal microscopy and, for the first time, detected a transportation process of Hst1: F-Hst1 (in red) was entrapped into an endocytic vesicle that pinched from the cell membrane (PKH labeled in green), which moved towards and associated with Hst-1-enriched mitochondria-like structure (Fig. 3). This finding clearly indicate that the uptake and subcellular targeting of F-Hst1 were mediated by endocytosis.

To further illustrate the relationship between endocytosis and Hst1’s uptake and functions, we tried to map the specific endocytosis pathways for the uptake of Hst1 using several well-characterized inhibitors as shown in Table 2. Our results showed that only CYD and CPZ, the respective inhibitors of phagocytosis/macropinocytosis and CME, significantly reduced the uptake of Hst1 in the cell to 35.3% and 20.6% respectively (Fig. 4a, b). In addition, amiloride, the specific inhibitor of micropinocytosis did not significantly influence the Hst1-cell interaction. These results suggest that phagocytosis and CME were responsible for the uptake of Hst1 in the adopted epithelial cells. Moreover, the promoting effect of Hst1 on cell metabolic activity could be completely abolished by either cytochalasin D or chlorpromazine (Fig. 4d), which indicated the critical role of sufficient uptake of Hst1 to activate mitochondria and cell metabolic activity. On the other hand, CPZ but not CYD dramatically reduced the Mander’s overlap coefficient from 0.88 to 0.33 (< 0.5), which indicates that CYD and CPZ differentially influenced the subcellular targeting of Hst1 and CPZ completely destroyed the co-localization between Hst1 and mitochondria (Fig. 4c). In addition, the other inhibitors (genistein, methyl-β-cyclodextrin, amiloride) did neither affect the uptake and subcellular targeting of Hst1, nor its stimulating effect on the cellular metabolism (Fig. 4d). These findings suggest that the CME was critical for the subcellular targeting of F-Hst1. These findings are indicative of our future study using a molecular tool, such as siRNA, shRNA, and CRISPR to provide further information to corroborate our hypothesis.

Following internalization, the endocytic vesicle is targeted to early endosomes where the cargo proteins are sorted to a variety of intracellular destinations.^34,35^ Therefore, we further tracked the whole intracellular trafficking process of Hst1 and demonstrated its tempo-spatial interaction with endosome network. We first assessed the spatial distributions of F-Hst1 and EE (indicated by GFP-fused Rab5) or LE (indicated by GFP-fused Rab7) within cells. In this study, we adopted a commercial gene transfection construct to express GFP fused to Rab5a in living cells, providing accurate and specific targeting to cellular EE-GFP. Concerns may be raised about the specificity of Rab5 to EE since Rab5 may also be localized in other cell organelles.^36^ EEA1 is a more EE-specific biomarker. In future study, a gene transfection construct to express GFP-EEA1^37^ should be adopted to further illustrate the temporospatial relationship between Hst1 and EE. In our confocal images, within a spread cell two areas are distinguished: cell body area and peripheral lamella area. As we recently reported,^9^ Hst1 is quickly targeted to mitochondria and forms a high-fluorescence-intensity area in the perinuclear area of the cell body, which we named as Hst1-enriched cell body area. Furthermore, we also detected that F-Hst1 was also enriched in a radial and linear distribution in the lamella area at a much lower fluorescence intensity (Fig. 5a), which might probably be attributed to the physical crosslinking between Hsts and G-/F-actin.^38^ This area was not detected in our previous study^9^ since its fluorescence intensity was much lower than that of the Hst1-enriched cell body area. Interestingly, there was a F-Hst1-barren area found between these two areas, forming the space for F-Hst1’s trafficking and targeting to mitochondria. Our images show that EE was mainly distributed in the F-Hst1-enriched cell body area and a small part was distributed near the border of the cell body in the F-Hst1-barren cell body area. In contrast, LE was distributed mainly in the Hst1-enriched cell body area. In the F-Hst1-barren cell body area, we found that F-Hst1 significantly co-localized with EE but not with LE (Fig. 5c). Such a phenomenon was in line with our previous finding that nearly no co-localization was found between F-Hst1 and lysosomes.^9^ Moreover, using the time-lapse imaging function of the confocal microscope, we found an approaching, touching and overlapping process between EE and F-Hst1 (Fig. 7a, b). These findings suggest that F-Hst1 interacted with EE for its intracellular trafficking, whereas it was not sorted to LE or lysosomes for degradation.

The targeting mechanism of endocytosed particles to mitochondria can be either EE-independent or EE-dependent. The former has recently been reported for the direct trafficking of styryl pyridinium FM dyes through CME to mitochondria, which is not influenced by either a dominant-negative or constitutively-active Rab5.^35^ The EE-dependent pathway is characterized by the transient “kiss and run” interactions between endosomes and mitochondria, which is found to mediate the direct iron transfer in erythroid cells (endosome–mitochondria interactions are modulated).^39^ In our study, we also found that F-Hst1-containing Rab5-labeled EE moved towards, interacted with and disassociated with mitochondria-like structure, during which F-Hst1 was released to mitochondria. Also considering the important role of Rab5 in Hst1-cell interaction,^6^ the intracellular targeting of F-Hst1 to mitochondria was more likely mediated through the “kiss and run” endosome–mitochondria interaction (Fig. 8). Such a delivery pattern seems to be associated with the low density of Rab5 in endocytic vesicles that loosely interact with EEA1-enriched endosomes and move towards the perinuclear area.^40^ Besides, microtubule- and actin-based motility of endosomes are frequently coordinated through Rab 5.^41–45^

Our previous study also showed that the inhibitor (U0126) of p-ERK but not p38 significantly reduced Hst1-induced migration of epithelial cells.^7^ Torres et al further confirm that Hst1 induces a biphasic activation of ERK1/2,^6^ which may partially be mediated by the arrestin scaffolding of the non-receptor tyrosine kinase c-Src.^29^ Interestingly, the thereby activated ERK1/2 is retained in the cytosol and neither translocates to the nucleus nor causes proliferation.^46^ These molecular events and functional characteristics of GPCR-activated ERK largely resemble Hst1’s functions*,* e.g., (1) U0126—the inhibitor of ERK activity abolished the promoting effect of Hst1 on cell migration^7,14^; and (2) Hst1 did not promote cell proliferation but cell metabolic activity.^9^ In our current study, we further show that U0126 significantly downregulates the Hst1’s uptake and promoting effect on cell metabolic activity, which indicates that the inhibition of ERK reversely suppresses Hst1’s endocytosis and subsequent functions. The underlying mechanisms, however, remain unveiled. One possible mechanism can be that the inhibition of endosomal ERK activity by U0126 hinders the trafficking and recycling of endosomes.^47^ Besides, the inhibition of mitochondria-localized ERK^48–52^ leads to a decrease in ATP levels,^48^ which may reversely influence the energy-dependent endocytosis and thus Hst1 signaling. Further experiments are highly needed to investigate and unravel the molecular mechanisms regulating Hst1’s endocytosis, trafficking, targeting, and promoting effect on cell metabolic activity.

Hitherto, the mechanism for Hst1’s promoting effect on the total cell metabolic activity remains largely unknown. Since Hst1 does not promote cell proliferation,^9^ we hypothesized that it promotes mitochondrial bioenergetics. We have recently adopted a Seahorse® XF96 analyzer to assess the cellular oxygen consumption rates (OCR), which shows that Hst1 substantially increases ATP production, basal OCR and maximal OCR in live cells (Data not shown). These data indicate that Hst1 significantly enhances mitochondrial energy metabolism in living cells. One possible mechanism to enhance the mitochondrial energy metabolism is to increase mitochondria-ER contacts (MERCs) (also referred to mitochondria-associated membranes (MAMs)), a highly important and dynamic hub controlling mitochondrial metabolism, calcium/Redox signaling, and lipid metabolism.^53^ For the aspect of bioenergetics, MERCs enable the transfer of high-concentration Ca^2+^ from ER to mitochondria,^54^ which promotes the activity of Krebs cycle’s dehydrogenases and ATP synthesis.^55^ Moreover, in our previous study, we have shown that Hst1 exhibits high co-localization coefficients not only with mitochondria but also with ER,^9^ which also suggests that Hst1 enhances MERCs. In the current study, we further provide more substantial evidence by showing that Hst1 indeed significantly promotes the co-localization of mitochondria and ER (Fig. 9). This result suggests that Hst1 enhances the formation of MERCs, thereby promoting mitochondrial energy metabolism.^56^

Although the target organelles of Hst1 are well established, the binding proteins of Hst1 associated with mitochondria and ER remain largely unknown. A very recent study shows that ER protein S2R/TMEM97 is one of the targets of Hst1.^16^ In that study, surface plasmon resonance analysis shows a direct binding of Hst1 to S2R/ TMEM97. Co-immunoprecipitation assay further shows that Hst1 binds to S2R/TMEM97 in cells. The siRNA KD of S2R/TMEM97 substantially compromised the in vitro pro-migratory effect of Hst1. Inspired by these results, we further investigated the effect of S2R/TMEM97 on Hst1’s uptake, subcellular targeting and promoting effect on cell metabolic activity. We showed that the siRNA KD of S2R/TMEM significantly reduced the uptake and mitochondria-targeting property (Fig. 10). KD of S2R/TMEM97 also nullified the promoting effect of Hst1 on cell metabolic activity. These data indicated that the ER target protein S2R/TMEM97 of Hst1 also critically regulated the Hst1-cell interaction. In fact, S2R/TMEM97 has been recognized as a cell metabolic regulator based on the fact that its specific ligand CM764 increases cellular Ca^2+^ transfer, total NAD^+^/NADH levels and the ATP levels and its specific antagonist significantly reduces cellular metabolic activity.^57^ Similarly, it is conceivable that the binding of Hst1 to S2R/TMEM97 increases mitochondrial energy production, which, in turn may further contribute to uptake, subcellular targeting and cell-activating effect of Hst1. Further studies are warranted to uncover how S2R/TMEM97 is involved in MERCs and how Hst1-mediated formation of MERCs affects cellular function and homeostasis.

The question may be raised whether the current findings can be extrapolated to other cell types. In our previous study,^9^ we have already shown that Hst1 is also targeted to mitochondria and ER not only in HO1N1 epithelial cells, but also in HaCaT human keratinocyte cells and primary human gingival fibroblasts. Moreover, Hst1 also significantly enhanced cellular metabolic activity of all three types of cells. In fact, in previous studies from both our group and other researchers, it has been shown that Hst1 shows a series of cell-activating effects on a large variety of cells, such as epithelial cells, fibroblasts, pre-osteoblasts endothelial cells.^8–10,12,58–61^ Therefore, the action pattern of Hst1 seems to be cell-type independent. Further studies should be performed to corroborate the current findings in more cell types, especially in the human primary cells.

## Conclusions

**Fig. 11:**
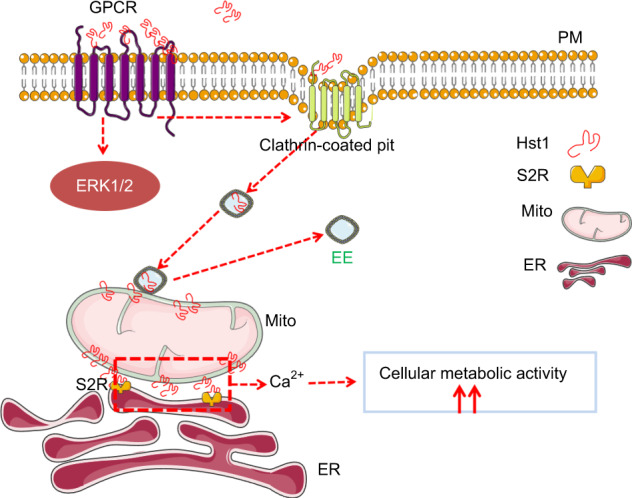
A schematic representation of the molecular mechanisms regulating Hst1’s endocytosis, trafficking, targeting and promoting effect on cell metabolic activity. Hst1 binds to GPCR, is then taken up via clathrin-meditated endocytosis, and subsequently, the Hst1-containing EEs moved towards the center of the cell, interacted and disassociated with mitochondria, during which F-Hst1 was released to mitochondria. Hst1 increases mitochondria-ER contacts (MERCs), which may enable the transfer of Ca^2+^ from ER to mitochondria to enhance the cellular metabolic activity. The ER target protein S2R/TMEM97 of Hst1 also critically regulates the Hst1-stimulating cellular metabolic activity

In conclusion, Hst1 was taken up by cells through an active and energy-dependent endocytosis. The specific inhibitors of GPCR, ERK and phagocytosis significantly reduced the uptake but not subcellular targeting of Hst1, which was accompanied by the nullification of the promoting effect of Hst1 on cell metabolic activity. In contrast, the specific inhibitor for CME and siRNA KD of S2R/TMEM97 significantly downregulated both the uptake and mitochondria-targeting of Hst1, which also abolished Hst1’s promoting effect on cell metabolic activity. By adopting a time-lapse imaging, we further illustrated the intracellular distribution pattern, trafficking and targeting process of Hst1, during which EE plays an important role. Taken together, our data show GPCR/endocytosis/ERK signaling/S2R was involved in the regulation of the internalization, mitochondria-targeting and -activating properties of human salivary Hst1 (Fig. 11).

## Materials and methods

### Solid-phase peptide synthesis

The peptides (Table 1) were manufactured by solid-phase peptide synthesis using 9-fluorenylmethoxycarbonyl (Fmoc)-chemistry on a SyroII synthesizer (Biotage, Uppsala, Sweden), essentially as published earlier.^62^ Fluorescent-labeled Hsts (F-Hst) were manufactured on resin using the fluorescent dye ATTO-647 (A647; ATTO-TEC, Siegen, Germany) as described previously.^9^ In previous studies, we have shown that the linkage of a large fluorescent molecule, e.g., Fluorescein isothiocyanate (FITC) to Hst1 does not influence the stimulating effect of Hst1 on cell migration.^7^ Besides, a biotinylated variant of Hst1 where biotin was linked to the lysine 17, displayed a comparable activity as the native peptide.^62^ These results show that the introduction of a large fluorescent molecule or bulky biotin group had no adverse effects on the biological activity, and exhibited similar biological and cell-binding activities compared to the unlabeled parent peptide.

**Table 1:** Amino acid sequences of Hst1 and its fluorescently labeled variants (F-)

Peptides	Amino acid sequences
(F-) Hst1	DSHEKRHHGYRRKFHEKHHSHREFPFYGDYGSNYLYDN
(F-) D-Hst1	dshekrhhgyrrkfhekhhshrefpfygdygsnylydn
(F-) Hst1^scr^	SDHSRHEEFKPRFHYHGGDYYRGRSKNFYHLEYKDHNH

Double underlined K/k indicates the amino acid to which the fluorescent dye ATTO-647 was coupled. D-enantiomer of Hst1 (D-Hst1); scrambled Hst1 (Hst1^scr^)

All peptides were purified by preparative reversed-phase high-performance liquid chromatography (RP-HPLC), using Dionex Ultimate 3000 system (Thermo Scientific, Breda, Netherlands) and a Grace Spring column (250 mm × 25 mm; Grace, Deerfield, IL, USA) containing 10 µm C18 TP beads (Vydac, Hesperia, CA, USA). Elution was performed with a linear gradient from 15 to 45% acetonitrile containing 0.1% TFA at a flow rate of 20 mL per min for 20 min. The absorbance of the column effluent was monitored at 214 nm, and peak fractions were pooled and lyophilized. Reanalysis by RP-HPLC on an analytic C18-column (218MS54; Vydac) developed with a similar gradient at a flow rate of 1 mL per min revealed a purity of at least 95%. The authenticity was confirmed by mass spectrometry with a Microflex LRF matrix-assisted laser desorption ionization time of flight mass spectrometer, equipped with an additional gridless reflectron (Bruker Daltonik GmbH, Bremen, Germany), as described previously.^62^

### Cell culture

The human buccal epithelial carcinoma cells line HO1N1 was obtained from the Japanese Collection of Research Bio-resources (Osaka, Japan). Cells were cultured in Dulbecco’s modified Eagle’s medium supplemented with Nutrient Mixture F-12 (DMEM/F-12, Gibco, Thermo Fisher Scientific, Waltham, MA, USA). The media were supplemented with 10% fetal calf serum (FCS; Invitrogen, Carlsbad, CA, USA), penicillin (10 units per mL), and streptomycin (10 μg·mL^−1^) (Invitrogen, Thermo Fisher Scientific). Cells were cultured in 75 cm^2^ cell culture flasks at 37 °C in a 5% CO_2_ atmosphere until near confluence, detached with 0.25% trypsin-EDTA (Invitrogen), counted in a hemocytometer, and seeded into new flasks or multi-well plates at the required cell densities.

### Treatments with chemical inhibitors

To analyze the potential mechanism on uptake pathways of Hst1, HO1N1 cells were pre-incubated with five types of endocytic inhibitors including 5 μmol·L^−1^ cytochalasin D (CYD, inhibitor of phagocytosis, Sigma–Aldrich, St. Louis, MO, USA), 25 μmol·L^−1^ chlorpromazine (CPZ, inhibitor of CME, Thermo Fisher Scientific), 50 μmol·L^−1^ amiloride (AMR, inhibitor of macropinocytosis mediated endocytosis, Thermo Fisher Scientific), 100 μmol·L^−1^ genistein (GEN, inhibitor of caveolae-mediated endocytosis, Thermo Fisher Scientific) and 250 μmol·L^−1^ methyl-β-cyclodextrin (MβCD, inhibitor of lipid raft mediated endocytosis, Thermo Fisher Scientific).

Energy dependence experiments were performed by pre-incubating the cells at 4 °C for 60 min to minimize cellular activity. Furthermore, cells were incubated with energy inhibitors including 20 μmol·L^−1^ sodium azide (NaN_3_, inhibitor of cytochrome C oxidase) and 50 μmol·L^−1^ ouabain (inhibitor of Na^+^/K^+^-ATPase) at 37 °C for 60 min.

Experiments on the dependence of GPCR were performed by pre-incubating the cells for 18 h with 100 ng·mL^−1^ Pertussis toxin (PTx), which catalyzes the ADP-ribosylation of the Gα_i_ subunits (Gα_i_, Gα_o_, and Gα_t_ except Gα_z_) of the heterotrimeric G protein, preventing the G proteins from interacting with G protein-coupled receptors on the cell membrane. To check the potential role of activated ERK signaling in the internalization and subcellular targeting of Hst1, we adopted U0126 that specifically inhibit the activation of ERK1 by inhibiting the kinase activity of MAP Kinase Kinase (MAPKK or MEK1/2).^63^ The cells were pre-incubated in the serum-free medium containing 10 μmol·L^−1^ U0126 for 60 min and then subjected to the medium with or without 10 μmol·L^−1^ Hst1. p38 MAPK dependence experiments were performed by pre-incubating the cells with inhibitor 10 μmol·L^−1^ SB203580 (SB), a potent inhibitor of α and β isoforms of p38 MAPK. All the inhibitors are summarized in Table 2.

**Table 2:** The specific inhibitors for endocytic pathways

Full name	Abbreviation	Targeted endocytic pathway	Mechanisms
Cytochalasin D	CYD	Phagocytosis and Macropinocytosis	Mycotoxin that binds actin and blocks its polymerization^65^
Chlorpromazine	CPZ	Clathrin-mediated endocytosis	AP2 inhibitor; blocks endosome recycling^66^
Amiloride	AMR	Macropinocytosis	Na^+^/H^+^ exchanger pump inhibitor^67^
Genistein	GEN	Caveolae-mediated endocytosis	Inhibition of recruitment of dynamin II^68,69^
Methyl-β-cyclodextrin	MβCD	Lipid-raft mediate endocytosis	Cholesterol depletion^70^

*CYD* cytochalasin D, *CPZ* Chlorpromazine, *AMR* amiloride, *GEN* genistein, *MβCD* methyl-β-cyclodextrin

After these pre-treatments 2 μmol·L^−1^ F-Hst1 was added to the culture medium and incubated for 60 min. The cells were then washed three times with Dulbecco’s phosphate-buffered saline (DPBS; Invitrogen) before microscopical analysis.

### Transfection/ KD of TMEM97

HO1N1 cells were grown in 8-well μ-Dishes (Ibidi GmbH, Munich, Germany) at a density of about 1 × 10^4^ cells per well. HO1N1 cells were transfected with reaction mixtures consisting of 12.5 pmol of a pool of 3 target-specific human small interfering RNAs (siRNA) composed of 19-25 nucleotides against TMEM97 (sc-93890, Santa Cruz Biotechnology, Dallas, TX, USA) for 48 h and 0.625 μL of Lipofectamine 2000 (11668027, Invitrogen, Carlsbad, CA, USA) in DMEM/F-12 media (Gibco, Thermo Fisher Scientific, Waltham, MA, USA). Complexes were incubated for 20 min at 24 °C and then added to the cells at 37 °C for an incubation continued for 24–48 h at 37 °C in 5% CO_2_. S2R/TMEM97 KD was confirmed with RT-PCR using TMEM97 primers (sc-93890-PR, Santa Cruz Biotechnology, Dallas, TX, USA).

### Hst1 uptake studies using confocal microscopy

HO1N1 cells were grown in 10 mm μ-Dishes (Ibidi GmbH, Munich, Germany) at a density of about 1 × 10^4^ cells per well, and treated with the various inhibitors as described above. To track the uptake pathways, 2 μmol·L^−1^ F-Hst variants were added to the cells. After 60 min of incubation, the cells were washed with DPBS. The acquisition of triple staining was done using a confocal laser scanning microscopy (CLSM) system (TCS SP8, Leica, Wetzlar, Germany), equipped with ×63 oil objective, with a pinhole size of 1 AU, (arbitrary unit), Z-step: 0.15 µm. The No. 20 Z-slice from the bottom (approximately 50 Z-slices per cell) was chosen since this slide contained the largest cross-section of nuclei. The intracellular fluorescence intensity of F-Hst1 was analyzed using Fiji software. First, the cell area was selected by using freeform selection tools, and then the intracellular fluorescence intensity of F-Hst1 was calculated by measuring the mean gray value of the selected area. The effects of various inhibitors on the uptake pathway of the F-Hst1 were evaluated by comparing the intracellular fluorescence intensity in the presence and absence of inhibitors. For each comparison, the experiments were performed on the same day in the same conditions and subsequently by using the same microscopic parameters for imaging. Fiji software was used for quantification.^64^

### Live cell imaging of membrane binding and subcellular trafficking of Hst1

HO1N1 cells were grown in 10 mm μ-Dishes (Ibidi GmbH) at a density of about 1 × 10^4^ cells per well. Prior to imaging, nuclear DNA was stained with NucBlue™ live cell stain according to the manufacturer’s protocol (Life Technologies, Grand Island, NY, USA). The cell membrane was labeled using PKH67 Green Fluorescent Cell Linker Kit (Sigma–Aldrich), according to the manufacturer’s instructions. Early endosome (EE) and late endosome (LE) were labeled using Invitrogen™/CellLight™ Early Endosomes-GFP, BacMam 2.0 (C10586, Invitrogen) and Invitrogen™/CellLight™ Late Endosomes-GFP, BacMam 2.0 (Invitrogen C10588, Invitrogen), following the manufacturer’s protocols. The staining solution was replaced with culture media and cells were immediately imaged. Confocal stacks were achieved with CLSM system (TCS SP8, Leica) using a 63 × 1.4 NA oil objective, and processing was completed using a 5% CO_2_ incubator at 37 °C. Time-lapse images of the living cells were recorded every 5 s. Fiji software was used for quantification.^64^

### Subcellular localization of the F-Hst1 using various inhibitors

Before imaging, nuclear DNA was stained with NucBlue™ live cell stain according to the manufacturer’s protocol (Invitrogen). For detection of mitochondria and ER, cells were washed with DPBS and incubated with 500 nmol·L^−1^ MitoTracker^®^ Green FM (Invitrogen, Carlsbad, CA, USA) and 500 nmol·L^−1^ ER-Tracker Blue-White DPX (Invitrogen, Carlsbad, CA, USA) for 30 min following the manufacturer’s protocol. The staining solution was replaced with cell culture media and cells were immediately imaged. Confocal stacks were achieved with CLSM system (TCS SP8, Leica) using a 63 × 1.4 NA oil objective. The confocal images were taken directly after incubation with F-Hst1 for 60 min. Image processing was performed in Fiji. Co-localization quantification of fluorescence signal was performed using the Fiji Coloc2 co-localization plugin (http://imagej.net/Coloc_2). In Coloc2, Mander’s overlap coefficients will vary from 0 to 1, the former corresponding to non- overlapping staining and the latter reflecting 100% co-localization of staining in both images.

### Cellular metabolic activity

We evaluated the influence of Hst1 and various inhibitors on cellular metabolic activity with the resazurin-based PrestoBlue reagent according to the manufacturer’s instructions (Invitrogen). HO1N1 cells were seeded into 96-well plates at a density of 1 × 10^4^ per well, in 100 μL culture medium, and incubated for 24 h to allow cell adherence. Cells were then incubated in the presence or absence of various inhibitors and Hst1 for 60 min. Untreated cells were used as control. The PrestoBlue solution (10 μL) was added into each well after 30 min, after which absorbance was measured at wavelengths 570 nm excitation and 600 nm emission. Data were presented as fold difference in OD (optical density) of absorbance by normalizing the OD value in other groups to that of the respective control group.

### Statistical analysis

All quantitative data in this study represent the mean value ± standard deviation (SD) for *n* ≥ 3 (number of experiments). Significance levels were determined by t test or one-way analysis of variance (ANOVA) unless explicitly stated otherwise (GraphPad Prism 9).

## Data Availability

The datasets used and/or analyzed during the current study are available from the corresponding author on reasonable request.
